# Microfluidics-generated PLA nanoparticles: impact of purification method on macrophage interactions, anti-inflammatory effects, biodistribution, and protein corona formation

**DOI:** 10.1039/d4pm00233d

**Published:** 2024-11-26

**Authors:** Jacob R. Shaw, Radha Vaidya, Fanny Xu, Shruti Dharmaraj, Ryan M. Pearson

**Affiliations:** a Department of Microbiology and Immunology, University of Maryland School of Medicine 685 W. Baltimore Street Baltimore MD 21201 USA; b Department of Pharmaceutical Sciences, University of Maryland School of Pharmacy 20 N. Pine Street Baltimore MD 21201 USA rpearson@rx.umaryland.edu +410-706-3257; c Marlene and Stewart Greenebaum Comprehensive Cancer Center, University of Maryland School of Medicine 22 S. Greene Street Baltimore MD 21201 USA

## Abstract

Polymeric nanoparticles (NPs) are traditionally formulated using batch methodologies that are poorly scalable and require time consuming, hands-on purification procedures. Here, we prepared poly(lactic acid) (PLA)-based polymeric NPs using a scalable microfluidics-based method and systematically investigated the impact of purification method (centrifugation *versus* tangential flow filtration (TFF)) to remove poly(vinyl alcohol) (PVA) on macrophage uptake, anti-inflammatory effects, biodistribution, and protein corona formation. TFF purification demonstrated significantly higher recovery of NPs compared to the centrifugation method, with little-to-no aggregation observed. PVA removal efficiency was superior with centrifugation, although TFF was comparable. NP cellular association, *in vitro* anti-inflammatory activity, and *in vivo* biodistribution studies suggested purification method-dependent alterations, which were correlated with protein corona profiles. This study underscores the potential of TFF, combined with microfluidics, as an efficient and high-yield purification method for NPs, and reveals the need for extensive confirmation of NP biological activity alongside physicochemical properties when developing NP therapeutics at-scale.

## Abstract 2

This study identifies TFF as an efficient method for scalable NP purification compared to centrifugation, though it alters biological properties that should be evaluated case-by-case.

## Graphical abstract

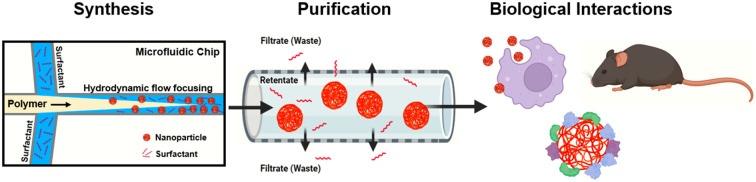

## Introduction

Nanoparticles (NPs) are a versatile class of materials that offer numerous advantages over traditional delivery systems. NPs stand out as adaptable drug delivery systems featuring advantageous characteristics such as extended circulation times, improved cellular uptake, reduced drug toxicity, and controlled release of cargo.^1,2^ Their recent surge in research interest stems from their versatility across diverse fields, with applications ranging from cancer treatment^3–5^ and vaccines,^6–9^ to combating infectious diseases.^10–12^ Notably, recent Food and Drug Administration (FDA) data indicates that, since 2016, several NP-based therapies (inorganic, polymeric, liposomal, and lipid-based) have been approved, underscoring the growing importance of NP-based formulations.^13,14^ Among these, polymeric NPs offer versatility and ease of design, making them suitable for applications such as vaccine delivery, antibiotic treatments, and cancer therapy.^15,16^ However, despite these promising attributes, a significant challenge remains. Non-scalable production and purification methods hinder the practical implementation of these promising NP technologies.^17,18^ Additionally, employing purification methods that do not alter the NP physicochemical properties and subsequent therapeutic efficacy is critical. Addressing these challenges is fundamental to fully harness the potential of NPs in complex healthcare issues and push the boundaries of medical science.

Two polymers that have been extensively used in NP formulations are poly(lactic acid) (PLA) and poly(lactic-*co*-glycolic acid) (PLGA) due to their biocompatibility and FDA Generally Recognized as Safe (GRAS) status. Several groups, including our own, have utilized these polymers for the preparation of NPs.^19–21^ Notably, our group has shown that PLGA and PLA NPs display inherently anti-inflammatory and immunomodulatory properties capable of treating severe inflammatory conditions.^19,22–25^ Polymeric NPs such as these can be prepared by desolvation, dialysis, ionic gelation, nanoprecipitation, solvent evaporation, salting out, spray drying, and supercritical fluid, but most commonly by oil-in-water (o/w) single emulsion solvent evaporation or nanoprecipitation.^15,26–28^ The advantage of solvent evaporation over the other techniques is that it utilizes ambient temperature and constant stirring. However, it is limited by higher NP polydispersity, larger particle sizes, batch-to-batch variations, low product recovery, and instability.^17,29^ These limitations can be addressed with alternative formulation methods to ensure reproducibility and scalable manufacturing. Microfluidic nanoprecipitation has emerged as a promising scaling-up method through its enhanced batch-to-batch reproducibility and reagent savings by enhanced loading and scalable formulation volumes.^17^ Microfluidic methods have been utilized for the reproducible synthesis of several types of NPs because of its precise control of flow parameters, controlled physical properties, particle size tunability, and scale-up potential.^24,29–31^ In the present study, microfluidics were employed as a scalable and reproducible synthesis method in conjunction with evaluating different NP purification strategies.

Based on the preparation method, impurities (*e.g.* solvents, surfactants, *etc*.) from NP formulation could be present in the final NP suspension that can alter the physicochemical characteristics of NPs and subsequent biological interactions.^32^ For these reasons, NPs are purified to maintain the quality and characteristics of the final NPs. Typically, centrifugation is used to pellet NPs and remove excess surfactant, however this method results in significant NP aggregation and reduced recovery.^24,32^ Furthermore, NP aggregation is associated with reduced blood circulation times and increased reticuloendothelial system (RES) clearance *in vivo*.^33^ Although centrifugation results in efficient surfactant removal, it is not feasible at a larger scale due to these limitations. Our goal was to develop a purification method to ensure optimal surfactant removal, improve NP recovery, and maintain nano-bio interactions compared to classical centrifugation. To avoid issues with the recovery of the NPs, tangential flow filtration (TFF) was investigated and optimized to remove the maximal amount of poly(vinyl alcohol) (PVA) surfactant from NP formulations. In the present study, we evaluated different TFF filters (300 kD and 750 kD) for the purification of microfluidics-generated PLA NPs. The physicochemical properties and stability of NPs before and after purification were compared to conventional centrifugation. Additionally, *in vitro* cellular association, *in vivo* organ distribution, and protein corona formation was evaluated. TFF proved to be an efficient purification technique that resulted in higher recovery and reduced NP aggregation as compared to centrifugation. However, the biological responses of TFF-purified NPs differed from those of centrifugation-purified NPs, highlighting the need for thorough validation of both biological activity and physicochemical properties when developing NP therapeutics at scale.

## Materials and methods

### Materials

The materials/chemicals used for the preparation of NPs by microfluidics were poly(lactic acid) (PLA) (approximate MW 11.3 kDa) with viscosity around 0.21 dL g^−1^ (lot number #1613-94) purchased from Lactel Absorbable Polymers (Birmingham, AL). Acetone, boric acid, iodine, potassium iodide, RPMI 1640, and poly(vinyl alcohol) (PVA) (MW 30 000–70 000, cat. no. P8136) were obtained from MilliporeSigma (St Louis, MO, USA). The following devices and materials were used for the purification of NPs by TFF [Minimate™ EVO Tangential Flow Filtration System (Pall Corporation, Port Washington, New York, USA)], Masterflex peristaltic pump (Cole Parmer Instrument Co., Vernon Hills, IL, USA), 300 kD and 750 kD MWCO membrane (MicroKros Hollow Fiber Filters, Spectrum Labs, Repligen, USA). Lipopolysaccharide (LPS) from *Escherichia coli* stereotype O111:B4, d-mannitol, sucrose, d-(+)-trehalose dihydrate were purchased from Sigma-Aldrich (St Louis, MO). Female C57BL/6 mice (5–6 weeks old) were purchased from University of Maryland Vet Resources (Baltimore, MD). Pooled healthy human plasma was gifted by Dr Maureen Kane at University of Maryland, Baltimore (Baltimore, MD).

### Preparation of nanoparticles by microfluidics

**Fig. 1:**
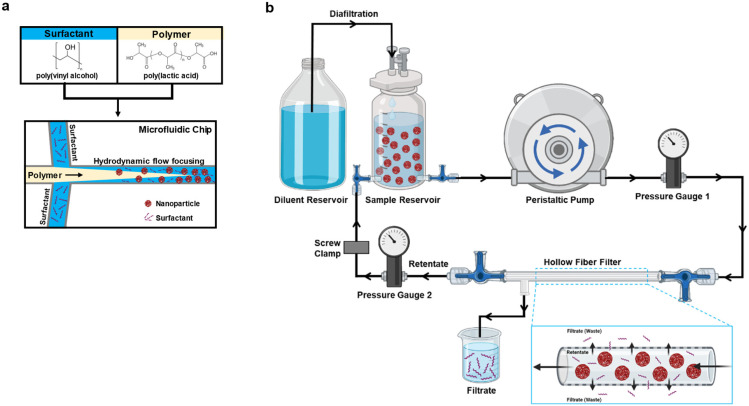
Schematic representation of the nanoparticle synthesis and purification. (a) Formulation of PLA–PVA nanoparticles using a flow focusing microfluidic device. (b) Schematic of the hollow fiber tangential flow filtration (TFF) system utilized for the purification of nanoparticles, shown under diafiltration. Figures were made using BioRender (https://www.biorender.com/).

The PLA–PVA NPs were prepared by nanoprecipitation using a microfluidic system (Dolomite, Royston, UK) and performed as described previously (Fig. 1a).^24^ The surfactant (0.1% PVA), polymer (1% PLA dissolved in acetone), and acetone were all filtered using a 0.2 μm filter to remove potential undissolved particulates that may clog the microfluidics tubing or chip prior to adding to the microfluidic system. The organic solutions were further degassed in a sonication bath. For nanoprecipitation, PLA and PVA flow rates were set at 1000 μL min^−1^ to generate a flow rate ratio (FRR) of 1. FRR was defined as the flow rate of the surfactant solution/flow rate of the polymer solution. Laminar flow at the microfluidic chip junction was continuously monitored using a Meros digital microscope to ensure stable nanoprecipitation of particles. Varying NP batch sizes were prepared (50 mg or 100 mg), collected into a beaker with constant magnetic stirring, and left overnight to allow for acetone evaporation. On the next day, the NPs were filtered using a 40 μm cell strainer and characterized for their physicochemical properties such as size, polydispersity index (PDI), and zeta potential using a Malvern Zetasizer as described.^4^ Fluorophore-labeled NPs (Cy5.5) were prepared by incorporating 0.5% w/v of a polymer–Cy5.5 conjugate into particles as described previously.^25^

### Washing of PLA–PVA NPs by centrifugation

Washing of PLA–PVA particles was performed by centrifugation. Briefly, NP suspensions were passed through a 40 μm cell strainer and chilled on ice for 15 minutes. The NPs were then centrifuged at 13 000*g* for 20 minutes at 4 °C and washed with autoclaved water. The centrifugation and washing steps were repeated thrice more. After the last wash, the NPs were filtered using a 40 μm cell strainer again. The size, polydispersity index, and zeta potential of the NPs were measured as described below. The NPs were lyophilized using cryoprotectant (4% sucrose, 3% mannitol solution). These NPs were then frozen at −80 °C before lyophilization using a Freezone 4.5 L −50 °C Complete Freeze Dryer System (Labconco, MO, USA) for two days.

### Purification of NPs to remove the surfactant by tangential flow filtration

The TFF system was set up as shown in Fig. 1b. Tangential Flow Filtration (TFF) was evaluated for the purification of NPs (Minimate™ EVO Tangential Flow Filtration System, Pall Corporation, Port Washington, New York). The pump used was Masterflex Easy-Load at a flow of 40 mL min^−1^. Transmembrane pressure was maintained between 10 and 20 psi using a tubing clamp. The prepared PLA–PVA NPs were poured into the reservoir/retentate capsule and diluted to a concentration of 0.5 mg mL^−1^ NPs with autoclaved water. 300 kD and 750 kD MWCO filters (MicroKros Hollow Fiber Filters, Spectrum Labs, Repligen, USA) with 20 cm^2^ surface area were used. The first mode used for the purification of the NPs was diafiltration. The feed reservoir was diluted with autoclaved water to maintain a constant volume. NPs were circulated through the TFF system using a peristaltic pump to allow removal of PVA. The NPs in the reservoir were dispersed with the help of a magnetic stirrer during the whole purification process. The system was run for 120 minutes and samples were collected at several time points from both the retentate and the filtrate (0, 20, 40, 60, 80, 100, and 120 min). While purifying fluorophore-labeled NPs, the system was covered with aluminum foil to avoid contact with light.

After diafiltration for 120 minutes, NP suspensions were concentrated by removing the water supplementation reservoir to prevent the replacement of removed solution. Once the volume dropped to less than 10 mL, NPs were recovered by redirecting the flow and flushing the system with an additional 10 mL of autoclaved water. The NPs after purification were evaluated for size, polydispersity index, and zeta potential. The purified NPs were also evaluated for their PVA content and lyophilized in 4% sucrose, 3% mannitol solution for later storage. Cleaning of the system was done using 200 mL 0.5 N NaOH followed by 500 mL of autoclaved water.

### PVA quantification

The PVA content was determined using a colorimetric method.^34^ The purified NPs obtained from the retentate were centrifuged at 13 000*g* for 5 minutes and 500 μL of supernatant was mixed with 300 μL of boric acid, 150 μL of water, and 50 μL of iodine–potassium iodide solution (0.05 M I_2_/0.15 M KI). A green color was formed between PVA and iodine in the presence of boric acid. The samples were incubated for one hour and higher PVA content was indicated by a darker color. Sample absorbance was measured in triplicate using a SpectraMax iD3 microplate reader at 690 nm and compared to a PVA standard curve. To quantify the amount of PVA in the filtrates, 500 μL of the filtrate was mixed in the same way as retentates, and the 690 nm absorbance was measured. NPs before and after purification were also analyzed for their PVA content.

### Physicochemical characterization using dynamic light scattering (DLS)

The *Z*-average size, polydispersity index (PDI), and zeta potential of the NPs were measured using a Malvern Zetasizer Nano ZSP (Malvern Panalytical; Cambridge, MA) as previously described.^24^ The NPs were dispersed in MilliQ water (pH 6) and particle measurements were performed at 23 °C at a scattering angle of 173°. For NPs post-lyophilization, lyophilized samples were reconstituted using MilliQ water, centrifuged at 13 000*g* for 5 minutes, the supernatant containing cryoprotectant was discarded, and the NP pellet was resuspended in MilliQ water for physicochemical characterization. All physicochemical properties were performed in triplicate with mean and standard deviation represented.

### Scanning electron microscopy (SEM)

The surface characteristics of NPs were studied using an FEI Quanta 200 (FEI, Hillsboro, OR). For these measurements, lyophilized NPs were adhered to an aluminum stub and sputter-coated with platinum and palladium at 20 mA for 20 s. Coated samples were loaded into the SEM instrument at a working distance of 9.5 mm and images were captured using a voltage of 15 kV.

### Animal studies

All animal procedures were performed in accordance with the Guidelines for Care and Use of Laboratory Animals of the University of Maryland, Baltimore and approved by the University of Maryland, Baltimore Institutional Animal Care and Use Committee (IACUC). Mice were housed under specific pathogen-free conditions in a facility managed by the University of Maryland, Baltimore Veterinary Resources.

### BMDM differentiation

Bone marrow-derived macrophages (BMDMs) were isolated from the tibias and femurs of 5–7 weeks-old female C57BL/6 mice following previously published methods.^35^ Briefly, complete RPMI 1640 media was prepared containing penicillin (100 units per mL), streptomycin (100 μg mL^−1^), 10% heat-inactivated fetal bovine serum (VWR, Radnor, PA), and 20% L929 cell conditioned medium (containing M-CSF) to induce BMDM differentiation. BMDMs were allowed to differentiate for 8 days, with cell conditioned media changes on days 3 and 6.

### Flow cytometry and inflammatory cytokine evaluation

BMDMs isolated from female C57BL/6 mice were seeded at 100 000 cells per well in a 24-well plate and treated with 10 μg mL^−1^ of Cy5.5-labeled NP samples. After NP treatment for 3 hours, 8 hours, or 24 hours, cells were washed with 1× PBS and lifted with Versene solution. Lifted cells were incubated with anti-CD16/32 antibody for FcR blocking before staining with anti-mouse CD11b-Pacific Blue (cat no. 101224, Clone M1/70, Biolegend) and F4/80-PE/Cyanine7 (cat no. 123114, Clone BM8, Biolegend). Cell viability was determined using Live/Dead Fixable Green from Biolegend. The stained cells were measured on a Cytek Aurora flow cytometer (Fremont, CA) and analyzed using FCS Express 7 De Novo Software (Glendale, CA).

Evaluation of cytokine production was performed as previously described.^24^ Briefly, BMDMs isolated from female C57BL/6 mice were seeded at 100 000 cells per well in a sterile 24-well plate. Cells were treated with 300 μg mL^−1^ of the different NP formulations for 3 hours at 37 °C and 5% CO_2_. Afterwards, cells were washed using 1× PBS to remove excess NPs, and LPS was introduced at 100 ng mL^−1^ in fresh L929-supplemented media. Cell culture supernatants were collected after 48 hours and analyzed using an enzyme-linked immunosorbent assay (ELISA) (Biolegend, San Diego, CA) to measure interleukin-6 (IL-6) following the manufacturer's protocols.

### Nanoparticle biodistribution study

Cy5.5-labeled PLA–PVA NPs were injected intravenously through the tail vein at 2 mg per female C57BL/6J mouse (6–8 weeks) (*n* = 3 per condition). Mice were then sacrificed after 3 hours; organs and plasmas were isolated for Xenogen *in vivo* imaging system (IVIS) analysis of organ distribution. Total radiant efficiency was calculated for all organs on a per-mouse basis and used to calculate the percent of NP amount in each organ.

### Protein corona evaluation

NPs suspended in 1× PBS at a concentration of 20 mg mL^−1^ were combined with 100% whole healthy human plasma at equal volumes, resulting in a final plasma concentration of 50%.^36,37^ Samples were allowed to incubate at 37 °C for 1 hour under slight agitation. After incubation, ice cold 1× PBS was added to preserve NP coronas, and unbound material was separated through centrifugation at 13 000*g*, 4 °C, for 30 minutes. Pelleted NPs were then washed three times through successive resuspension and centrifugation in ice cold PBS. Corona proteins were eluted from the final NP corona pellet by adding Laemmli buffer (0.277 M Tris-HCl, 44.4% Glycerol, 4.4% LDS, 2-mercaptoethanol, 0.02% bromophenol blue) and heating samples to 95 °C for 5 minutes. Denatured proteins were separated from NPs through centrifugation, and corona proteins were run through a 4%–15% gradient SDS-PAGE gel. Gels included a molecular weight protein ladder standard (product no. 1610394) from Bio-Rad (Hercules, CA). Thereafter, PAGE gels were stained with Coomassie and destained following manufacturer guidelines. Gels were imaged on ThermoFisher iBright imaging system and processed using ImageJ Version 1.54 h. Protein loading amounts were normalized to 0.5 mg of NPs per well.

### Statistical analysis

All the statistical analyses were performed using Prism 9 (GraphPad, San Diego, CA). Results are reported with mean and standard deviation (SD). Significant differences between multiple groups were determined by one-way or two-way ANOVA with Tukey *post hoc* test. In all cases, *p* < 0.05 was considered to be statistically significant.

## Results

### Microfluidic formulation of PLA–PVA NPs

**Fig. 2:**
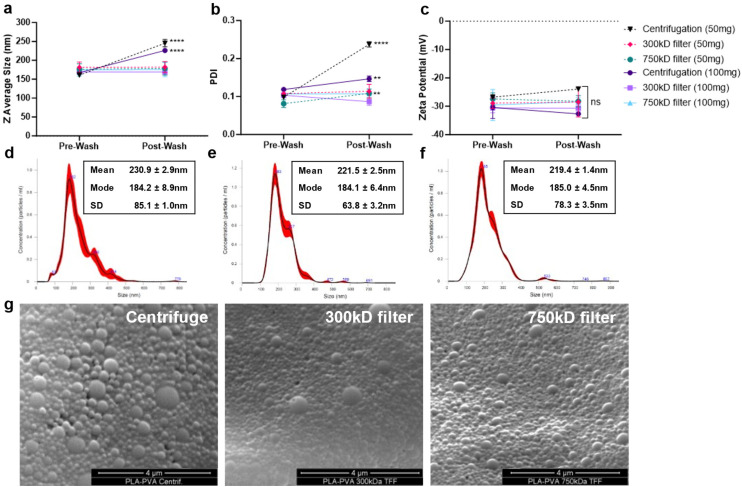
Nanoparticle physicochemical characterization after purification. (a–c) Nanoparticles physicochemical properties before and after purification by centrifugation, 300 kD filter TFF, or 750 kD filter TFF were characterized using dynamic light scattering (DLS). Nanoparticle tracking analysis (NTA) of 100 mg batches post-purification with (d) centrifugation, (e) 300 kD filter TFF, (f) 750 kD filter TFF. Mean and mode diameter is displayed along with size standard deviation. (g) SEM images of 100 mg batch size NP using the various purification methods.

NPs were formulated using microfluidics with a FRR of 1, PLA concentration of 1% w/v in acetone (10 mg mL^−1^), and PVA surfactant concentration of 0.1% in water (1 mg mL^−1^) (Fig. 1a). Based on our previous studies, microfluidic parameters were adjusted to formulate 200 nm NPs, with low PDI (<0.2), and negative zeta potential due to the carboxylic acid-terminated PLA polymer.^24^ Two different batch sizes (50 and 100 mg) were prepared that only varied by the time that the system was run. NP purification was then performed either using centrifugation or TFF to remove excess surfactant (Fig. 1b). Larger batch sizes were attempted for purification, but resulted in significant TFF filter clogging and would likely require a larger filter surface area cassette than currently used. Washing through centrifugation significantly increased the particle size and polydispersity index (PDI) indicative of NP aggregation as measured by dynamic light scattering (DLS) (Fig. 2a and b); however, purification of the NPs by TFF using either 300 kD or 700 kD MWCO filters showed a consistent size and polydispersity index following purification, with a minor increase in PDI for the 750 kD 50 mg batch. For all batches purified, the zeta potential post-wash was unchanged compared to the pre-wash (Fig. 2c). Nanoparticle tracking analysis (NTA) recapitulated the increased mean size and standard deviation (SD) of centrifuged NPs (Fig. 2d), while also displaying a more homogeneous size distribution profile for the TFF-purified NPs (Fig. 2e and f). Scanning electron microscopy (SEM) imaging of these NPs indicated similar spherical morphologies between the formulations, with TFF purified NPs showing more homogeneous size distributions (Fig. 2g).

### Quantification of surfactant removal from purified NPs

**Fig. 3:**
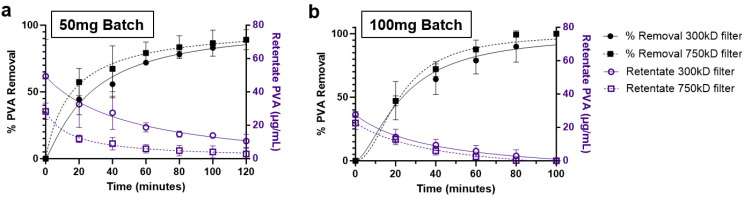
TFF PVA removal time-course. Quantified % PVA removal (black) and remaining PVA weight (blue) for (a) 50 mg and (b) 100 mg batches of PLA–PVA nanoparticles purified by TFF (300 kD and 750 kD filters).

Two methods were followed for the purification of the prepared NPs. One was washing of the NPs by centrifugation, and the other was by diafiltration through TFF. Two filters were evaluated for the TFF system, namely 300 kD and 750 kD MWCO. The transmembrane pressure for the system was maintained between 10–20 psi. These filters demonstrated logarithmic PVA removal over the course of 120 minutes under TFF diafiltration. Interestingly, both filters showed similar PVA removal trends, with the 750 kD filter demonstrating marginally faster PVA removal, reaching approximately 90% removal after 100 minutes (Fig. 3a and b). Quantification of the final PVA concentration in NP suspensions indicated that % PVA removal was better in the smaller NP batches (Table 1). Alternatively, washing of the NPs by centrifugation resulted in a slight improvement in PVA removal, however the final NP recovery was approximately 2 to 4-fold lower than TFF which achieved upwards of 78% recovery. To further validate overall particle concentrations were similar after purification, final suspensions for the 100 mg batches were normalized to 1 mg mL^−1^ NP and measured using nanoparticle tracking analysis (NTA). NP concentration increased with increasing final NP yield, meaning the minor excess in surfactant did not significantly reduce actual NP concentrations (Table 1).

**Table 1:** Purified NP yield and PVA removal. The concentration of the 50 mg NP batches was not determined (n.d.)

Sample	Batch size (mg)	% PVA removal	% NP yield	NP concentration (NPs per mL)
Centrifugation	50	99.9 ± 0.1	23.4 ± 12.4	n.d.
	100	99.3 ± 0.5	17.2 ± 5.3	1.16 × 10^−9^ ± 3.23 × 10^−7^
300 kD	50	95.2 ± 0.8	48.9 ± 14.8	n.d.
	100	92.9 ± 3.4	74.9 ± 11.2	1.26 × 10^−9^ ± 1.19 × 10^−7^
750 kD	50	96.5 ± 1.1	59.3 ± 12.2	n.d.
	100	87.6 ± 5.5	78.2 ± 10.7	1.30 × 10^−9^ ± 4.86 × 10^−7^

### Evaluation of NP-macrophage cellular interactions

**Fig. 4:**
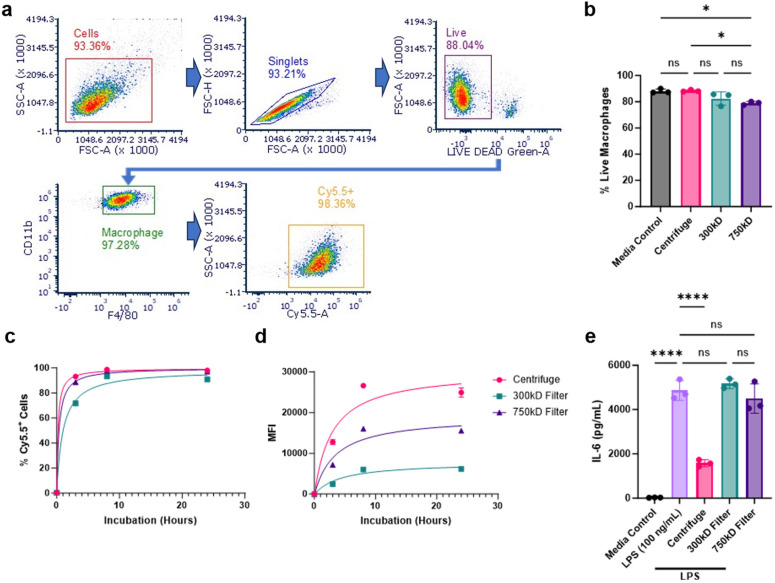
NP-cellular interactions post-purification. (a) Representative flow cytometry gating strategy of centrifuge-purified Cy5.5-labeled NPs incubated with BMDM cells after 24 hours. Arrows indicate parent gate used for subsequent graph analysis. (b) Percent cellular viability of all BMDM cells after a 24 hour incubation with NPs or media control. (c) Percent of Cy5.5-NP^+^ live BMDM cells over a 24 hour incubation, determined through flow cytometry. (d) Mean fluorescence intensity (MFI) of Cy5.5-NP^+^ live BMDM cells. (e) ELISA quantification of IL-6 cytokine secretions in the supernatant of BMDM cells treated with NPs and LPS for 48 hours. Significance was calculated using a one-way ANOVA with Tukey *post hoc* test. *P < 0.05; **P < 0.01; ***P < 0.001, ****P < 0.0001. ns, not significant (*P* > 0.05).

We next evaluated cellular interactions of these various NPs with bone marrow-derived macrophages (BMDM). BMDMs were treated with Cy5.5-labeled NPs from the three purification conditions and NP-cellular association was measured at 3, 8, and 24 hours using flow cytometry. 100 mg batch sizes were chosen for this and subsequent studies due to their superior NP yield and comparable surfactant removal. NP-cellular association was determined by measuring live/Cy5.5-NP^+^ macrophage populations using the classical macrophage surface markers CD11b and F4/80 (Fig. 4a). We additionally analyzed the percentage of total live cells after 24 hours of NP treatment. The 750 kD filter treatment group showed a slight reduction in viability (<10% from control) (Fig. 4b). All NP conditions showed a time-dependent cellular associations and achieved nearly 100% cellular association after 24 hours; however, 300 kD purified NPs demonstrated delayed uptake compared to the other NPs (Fig. 4c). Additional median fluorescence intensity (MFI) quantification of these cells, signifying the amount of Cy5.5-NPs associated per cell, further indicated that the 300 kD filter, along with the 750 kD filter, had reduced NP association compared to centrifuge NPs (Fig. 4d).

Given that we previously demonstrated PLA–PVA NPs purified through centrifugation possess inherent anti-inflammatory properties, we investigated whether differences in cellular association affects NP anti-inflammatory effects.^19,24^ To assess this, BMDM cells were first treated with the various NPs, stimulated with the pro-inflammatory molecule lipopolysaccharide (LPS), then IL-6 production was measured as a marker of inflammation using ELISA. Centrifuged PLA–PVA NPs were successfully able to prevent IL-6 secretion compared to the LPS control, however, both NPs purified through TFF did not significantly reduce inflammation (Fig. 4e). For this assay, NPs were administered to cells for 3 hours prior to LPS stimulation. Given the significant reduction in cellular uptake of TFF-purified NPs, particularly at earlier timepoints, the lack of IL-6 prevention is likely explained by TFF-purified NPs hindered ability to deliver sufficient amounts of intracellular lactic acid necessary for macrophage modulation and pro-inflammatory cytokine prevention.

### *In vivo* organ distribution

**Fig. 5:**
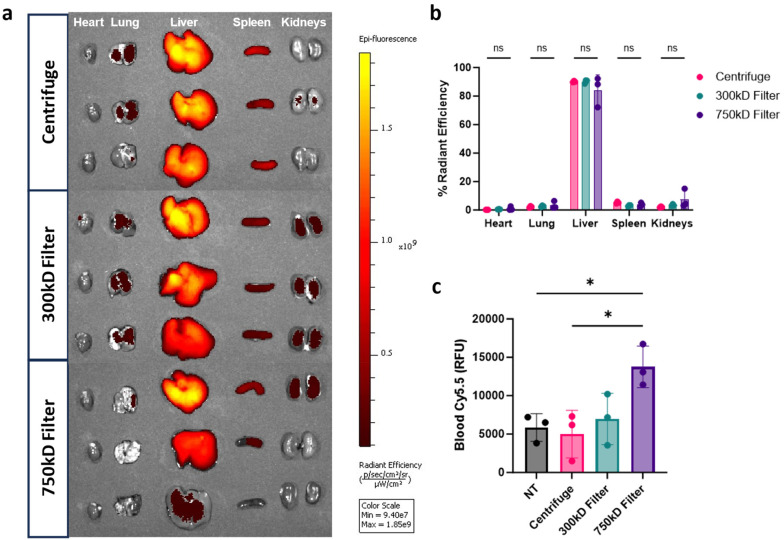
*In vivo* nanoparticle distribution. (a) Organ biodistribution of Cy5.5-labeled PLA NPs 3 hours post i.v. injection (2 mg dose) in naïve C57BL/6 mice. Heart, liver, lung, spleen, and kidneys were isolated and analyzed *via in vivo* imaging system (IVIS) for NP fluorescence. (b) Percent of total radiant efficiency is presented in the graph (*n* = 3 mice per group). (c) Cy5.5 fluorescence measured from whole blood taken at 3 hours post i.v. injection. Significance was calculated using a one-way ANOVA with Tukey *post hoc* test. *P < 0.05; **P < 0.01; ***P < 0.001, ****P < 0.0001. ns, not significant (*P* > 0.05).

Given the differences in BMDM cellular association *in vitro*, we assessed organ biodistribution of these NPs. Mice were injected intravenously (i.v.) with the Cy5.5-labeled PLA–PVA NP variants. After 3 hours, organs and blood were isolated to quantify NP biodistribution through Cy5.5 fluorescence. As expected, distribution was most prevalent in the liver, with less than 10% sequestering in the heart, lungs, spleen, and kidneys (Fig. 5a and b). Interestingly, there were no significant differences in organ distributions observed between the different purified formulations despite the reduction of macrophage associated *in vitro*. To measure residual circulating NPs, quantification of Cy5.5 fluorescence in whole blood indicated that 750 kD filter purified samples had higher residual circulating NPs compared to the other conditions (Fig. 5c).

### NP protein corona analysis

**Fig. 6:**
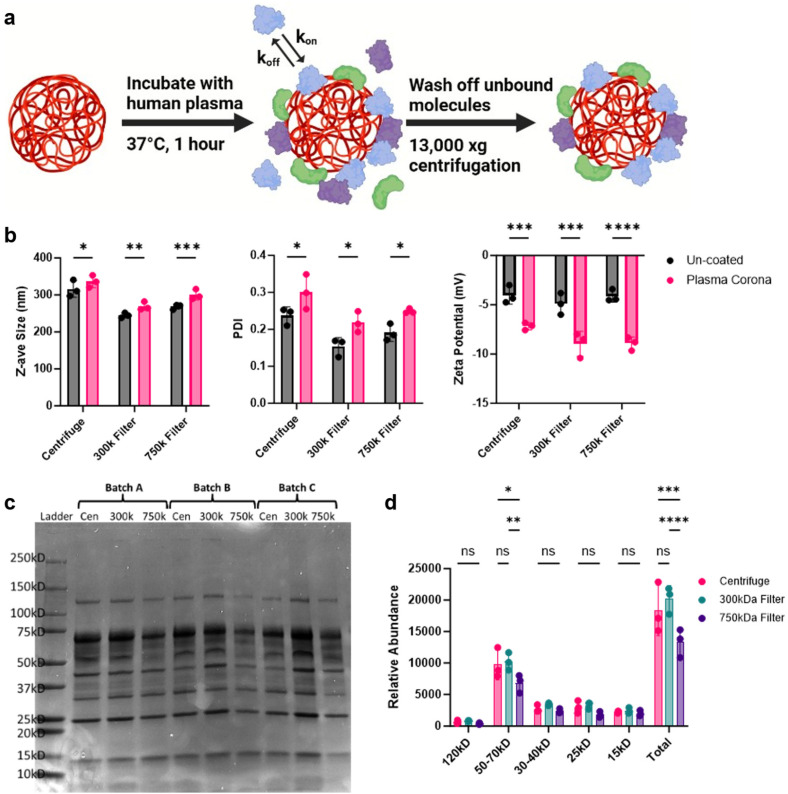
Purification-dependent NP protein coronas. (a) Schematic representation of NP protein corona formation. Figure was made using BioRender (https://www.biorender.com/). (b) Physicochemical characterization of NP protein coronas. (c) NP corona-bound proteins were eluted from human plasma coated PLA–PVA formulations and run through an SDS-PAGE gel, stained with Coomassie for total protein. (d) Protein band densitometry of the three batches was calculated using ImageJ. Significance was determined using a two-way ANOVA with Tukey *post hoc* test. Data sets are presented as mean ± S.D. (*n* = 3). *P < 0.05; **P < 0.01; ***P < 0.001, ****P < 0.0001. ns, not significant (*P* > 0.05).

When NPs are introduced into complex biological matrices, like blood, they rapidly adsorb biomolecules which transforms the NP synthetic identity to a new biological one.^38–40^ This coating, termed the protein corona, significantly influences the cellular recognition and biodistribution of NPs.^40,41^ Due to the potential increase in NP circulation times observed, we sought to measure the protein corona of these NPs. To achieve this, we coated the NPs with healthy human plasma and evaluated differences in protein fingerprints between the different purification methods (Fig. 6a). DLS analysis of these coated NPs showed an increase in size, PDI, and altered zeta potential, all indicative of protein corona formation (Fig. 6b). The corona-bound proteins were then eluted from the NPs and analyzed using SDS-PAGE to observe differences (Fig. 6c). Notably, NPs purified with the 750 kD filter displayed a marked reduction in protein band density compared to the other conditions. Further densitometry analysis confirmed that proteins in the 50–75 kD range were significantly less intense, along with a reduction in total protein content (Fig. 6d). This reduction in protein adsorption by the 750 kD-filtered NPs may be attributed to residual PVA surfactant, which could hinder protein binding and potentially be associated with the increased fluorescence signal in the blood.

## Discussion

Nanotechnology has emerged as a powerful tool in the development and enhancement of therapeutic formulations. Nanomedicines have shown promise in preventing and treating severe conditions such as cancer, cardiovascular diseases, and immunological disorders.^13^ In particular, NPs offer significant advantages for biological applications, both *in vitro* and *in vivo*, due to their ability to improve the solubility, stability, and controlled release of encapsulated drugs, while also reducing toxicity.^2^ Microfluidics-based generation of NPs has gained popularity in recent years for its reproducibility, controlled physical properties, and scalability compared to traditional bulk production methods.^29^ However, efficient purification of NPs, particularly in terms of surfactant removal and recovery, while maintaining physicochemical properties and biological activity, remains a significant challenge in nanotechnology.

In this study, we synthesized PLA–PVA NPs using microfluidics and purified them through TFF, comparing the results to conventional centrifugation in terms of physicochemical properties, macrophage interactions, anti-inflammatory effects, biodistribution, and protein corona formation (Fig. 1). While microfluidics provides precise control over physicochemical properties, NP performance *in vivo* depends on characteristics like size, polydispersity, and zeta potential, therefore, maintaining these properties is crucial to retain therapeutic efficacy.^42^ Our results show that NPs produced using microfluidics result in consistent size, PDI, and zeta potential yet this consistency can be undermined by poor purification procedures, like centrifugation (Fig. 2). Centrifugation is widely used for NP purification, effectively removing excess reactants like surfactants.^32^ However, it can significantly alter NP size distributions and lead to irreversible aggregation. Furthermore, centrifugation speed plays a crucial role, as different NP sizes are pelleted at different speeds, which can markedly change the characteristics of the final suspension.^43^ For example, lower centrifugation speeds pellet larger NPs, leaving smaller ones in suspension to be discarded. In contrast, TFF enables the purification of the entire NP suspension without bias towards a specific NP size. While TFF has been used for NP purification, its combination with microfluidics has been less explored. Previous research demonstrated that using 300 kD TFF filters for PLGA–PVA NPs, synthesized by batch emulsification, was either inefficient or required prolonged run times for adequate surfactant removal.^32^ In contrast, our study found that employing 300 kD and 750 kD filters with higher transmembrane pressures (10–20 psi) effectively removed 90–95% of PVA within 2 hours while maintaining NP sizes and PDI (Fig. 3, Table 1).

It is well-known that the biological fate of NPs depends on their interactions with blood cells and plasma biomolecules. Key factors such as particle size, surface charge, morphology, and surfactant presence play crucial roles in these interactions with the biological milieu.^44,45^ We demonstrated that NPs purified by TFF exhibited significantly reduced macrophage uptake *in vitro* compared to centrifugation, which was particularly evident by the drastic reduction in cellular MFI representing NP amount per cell (Fig. 4c and d). NP uptake plays a pivotal role in their inherent anti-inflammatory effects. Therefore, NPs purified by centrifugation resulted in a significant reduction in IL-6 cytokine secretions than those purified by TFF (Fig. 4e). This observation aligns with previous findings that the anti-inflammatory potential of PLA-based NPs is driven by internalization and intracellular release of lactic acid, which subsequently inhibits NF-κB and p38 MAPK phosphorylation.^19,23^ Thus, variations in the purification method were found to influence the bioactivity of the NPs by modulating their uptake profiles, potentially by maintaining residual surfactant coatings or preventing aggregation which is associated with increased phagocytic uptake and clearance. These results clearly demonstrate the need for extensive biological evaluation of NP formulations to be performed when critical process parameters are changed including method of NP fabrication and purification to ensure their desired activity is retained.

The reticuloendothelial system (RES), which primarily consists of mononuclear phagocytic cells circulating in the bloodstream and mature macrophages residing in the lungs, liver, and spleen, is a significant barrier to sustained pharmacokinetics after intravenous administration. NP organ distribution is primarily driven by size, where NPs larger than 200 nm tend to accumulate in the liver and spleen.^42^ Our *in vivo* results showed that, while overall NP organ distribution was unaffected, NPs purified with a 750 kD TFF filter remained detectable in the blood after 3 hours (Fig. 5). Despite the organ distribution being similar for all NP formulations, our *in vitro* cell association studies suggest that differences may arise on a cellular level as we have previously shown.^46^ Surface modification of NPs with surfactants has been reported to reduce interactions with blood components, thereby prolonging NP circulation time and reducing RES clearance.^47,48^ These differences in cellular uptake could be explained by the relatively higher residual surfactant seen in TFF purified samples preventing NP opsonization by blood components. To assess this, we formed protein coronas on NPs using healthy human plasma. Protein corona analysis revealed that 750 kD filter-purified NPs had significantly fewer adsorbed proteins in total and particularly in the 50 kD to 75 kD size range (Fig. 6). This size range in the coronas has primarily been associated with serotransferrin, complement factor fragments, and albumin, which could explain the significant differences in cellular association and blood clearance.^49,50^ However, in-depth mass spectrometry studies are necessary to quantitatively identify the precise NP corona compositions. Reduced NP opsonization is often linked to prolonged circulation times, a phenomenon researchers often strive to achieve by modifying the NP surface with substances like poly(ethylene glycol) (PEG) to prevent protein binding.^51^ Determining the mechanism by with 750 kD-purified NPs reduce protein corona formation could be beneficial for future therapeutics.

While we have shown that PLA–PVA NPs can be efficiently purified by TFF, the precise changes in their biological interactions relative to centrifugation are still unknown and could be influenced by residual surfactant. Additionally, future studies should consider other types of polymers, surfactants, and excipients, evaluate the purification parameters, and the subsequent biological interactions. Other filter sizes and surface areas could be studied for the purification system to understand alterations in surfactant removal based on these parameters. Along with the many advantages of the microfluidics system, some disadvantages are the limited solvent compatibility, chip clogging, and volumetric throughput.^52^ The system could be modified for better NP formulation scale-up by incorporating TFF from the microfluidic formulation. For this challenge, architectures should be developed to streamline microfluidic synthesis directly into TFF purification as a joint system.

## Conclusion

In the present study, we formulated polymer-based PLA–PVA NPs using a high-throughput microfluidic platform and evaluated their purification through conventional centrifugation or TFF. We tested two different TFF membrane molecular weight cutoffs and two NP batch sizes to evaluate surfactant removal and scale-up potential. Centrifugation led to NP aggregation, increased size and PDI, low product recovery, but effective surfactant removal. In contrast, TFF purification preserved consistent physicochemical properties, achieved comparable surfactant removal, and resulted in superior final product recovery. While increasing the TFF filter MWCO did not significantly affect yield or PVA removal, it extended NP blood concentration and reduced NP protein corona adsorption. Despite these differences, overall *in vivo* organ distribution did not significantly vary between purification methods. Our findings highlight the advantages of integrating high-throughput microfluidic NP synthesis with TFF purification, offering a more effective approach for NP recovery and maintenance of physicochemical properties. This integration can help enhance reproducibility, development speed, scale-up, and translation of future NP formulations.

## Author contributions

J. R. S., R. V. and R. M. P. conceived the project and wrote the manuscript. J. R. S., R. V., and F. X. designed and performed the *in vitro* experiments. J. R. S. and S. D. performed *in vivo* experiments and sample processing. J. R. S. performed computational and statistical analyses. R. M. P. acquired funding and supervised the project. All authors edited and approved the final manuscript.

## Data availability

Raw data from this study are available from the corresponding author (R. M. P.) upon reasonable request.

## Conflicts of interest

There are no conflicts to declare.

## References

[cit1] Patra J. K., Das G., Fraceto L. F., Campos E. V. R., Rodriguez-Torres M. D. P., Acosta-Torres L. S., Diaz-Torres L. A., Grillo R., Swamy M. K., Sharma S., Habtemariam S., Shin H.-S. (2018). Nano Based Drug Delivery Systems: Recent Developments and Future Prospects. J. Nanobiotechnol..

[cit2] Mitchell M. J., Billingsley M. M., Haley R. M., Wechsler M. E., Peppas N. A., Langer R. (2021). Engineering Precision Nanoparticles for Drug Delivery. Nat. Rev. Drug Discovery.

[cit3] Gavas S., Quazi S., Karpiński T. M. (2021). Nanoparticles for Cancer Therapy: Current Progress and Challenges. Nanoscale Res. Lett..

[cit4] Yao Y., Zhou Y., Liu L., Xu Y., Chen Q., Wang Y., Wu S., Deng Y., Zhang J., Shao A. (2020). Nanoparticle-Based Drug Delivery in Cancer Therapy and Its Role in Overcoming Drug Resistance. Front. Mol. Biosci..

[cit5] Cheng Z., Li M., Dey R., Chen Y. (2021). Nanomaterials for Cancer Therapy: Current Progress and Perspectives. J. Hematol. Oncol..

[cit6] Zhao L., Seth A., Wibowo N., Zhao C.-X., Mitter N., Yu C., Middelberg A. P. J. (2014). Nanoparticle Vaccines. Vaccine.

[cit7] Bezbaruah R., Chavda V. P., Nongrang L., Alom S., Deka K., Kalita T., Ali F., Bhattacharjee B., Vora L. (2022). Nanoparticle-Based Delivery Systems for Vaccines. Vaccines.

[cit8] Guerrini G., Magrì D., Gioria S., Medaglini D., Calzolai L. (2022). Characterization of Nanoparticles-Based Vaccines for COVID-19. Nat. Nanotechnol..

[cit9] Scotland B. L., Shaw J. R., Dharmaraj S., Caprio N., Cottingham A. L., Joy Martín Lasola J., Sung J. J., Pearson R. M. (2023). Cell and Biomaterial Delivery Strategies to Induce Immune Tolerance. Adv. Drug Delivery Rev..

[cit10] Gao W., Thamphiwatana S., Angsantikul P., Zhang L. (2014). Nanoparticle Approaches against Bacterial Infections. Wiley Interdiscip. Rev.: Nanomed. Nanobiotechnol..

[cit11] Lee N.-Y., Ko W.-C., Hsueh P.-R. (2019). Nanoparticles in the Treatment of Infections Caused by Multidrug-Resistant Organisms. Front. Pharmacol..

[cit12] Kirtane A. R., Verma M., Karandikar P., Furin J., Langer R., Traverso G. (2021). Nanotechnology Approaches for Global Infectious Diseases. Nat. Nanotechnol..

[cit13] Anselmo A. C., Mitragotri S. (2019). Nanoparticles in the Clinic: An Update. Bioeng. Transl. Med..

[cit14] Anselmo A. C., Mitragotri S. (2021). Nanoparticles in the Clinic: An Update Post COVID-19 Vaccines. Bioeng. Transl. Med..

[cit15] Zielińska A., Carreiró F., Oliveira A. M., Neves A., Pires B., Venkatesh D. N., Durazzo A., Lucarini M., Eder P., Silva A. M., Santini A., Souto E. B. (2020). Polymeric Nanoparticles: Production, Characterization, Toxicology and Ecotoxicology. Molecules.

[cit16] Beach M. A., Nayanathara U., Gao Y., Zhang C., Xiong Y., Wang Y., Such G. K. (2024). Polymeric Nanoparticles for Drug Delivery. Chem. Rev..

[cit17] Shepherd S. J., Issadore D., Mitchell M. J. (2021). Microfluidic Formulation of Nanoparticles for Biomedical Applications. Biomaterials.

[cit18] Herdiana Y., Wathoni N., Shamsuddin S., Muchtaridi M. (2022). Scale-up Polymeric-Based Nanoparticles Drug Delivery Systems: Development and Challenges. OpenNano.

[cit19] Casey L. M., Kakade S., Decker J. T., Rose J. A., Deans K., Shea L. D., Pearson R. M. (2019). Cargo-Less Nanoparticles Program Innate Immune Cell Responses to Toll-like Receptor Activation. Biomaterials.

[cit20] Lü J.-M., Wang X., Marin-Muller C., Wang H., Lin P. H., Yao Q., Chen C. (2009). Current Advances in Research and Clinical Applications of PLGA-Based Nanotechnology. Expert Rev. Mol. Diagn..

[cit21] Casalini T., Rossi F., Castrovinci A., Perale G. (2019). A Perspective on Polylactic Acid-Based Polymers Use for Nanoparticles Synthesis and Applications. Front. Bioeng. Biotechnol..

[cit22] Lasola J. J. M., Kamdem H., McDaniel M. W., Pearson R. M. (2020). Biomaterial-Driven Immunomodulation: Cell Biology-Based Strategies to Mitigate Severe Inflammation and Sepsis. Front. Immunol..

[cit23] Lasola J. J. M., Cottingham A. L., Scotland B. L., Truong N., Hong C. C., Shapiro P., Pearson R. M. (2021). Immunomodulatory Nanoparticles Mitigate Macrophage Inflammation via Inhibition of PAMP Interactions and Lactate-Mediated Functional Reprogramming of NF-κB and P38 MAPK. Pharmaceutics.

[cit24] Truong N., Black S. K., Shaw J., Scotland B. L., Pearson R. M. (2021). Microfluidic-Generated Immunomodulatory Nanoparticles and Formulation-Dependent Effects on Lipopolysaccharide-Induced Macrophage Inflammation. AAPS J..

[cit25] Truong N., Cottingham A. L., Dharmaraj S., Shaw J. R., Lasola J. J. M., Goodis C. C., Fletcher S., Pearson R. M. (2024). Multimodal Nanoparticle-Containing Modified Suberoylanilide Hydroxamic Acid Polymer Conjugates to Mitigate Immune Dysfunction in Severe Inflammation. Bioeng. Transl. Med..

[cit26] Krishnamoorthy K., Mahalingam M. (2015). Selection of a Suitable Method for the Preparation of Polymeric Nanoparticles: Multi-Criteria Decision Making Approach. Adv. Pharm. Bull..

[cit27] Cele T. , Preparation of Nanoparticles, in Engineered Nanomaterials - Health and Safety, IntechOpen, 2020

[cit28] Vauthier C., Bouchemal K. (2009). Methods for the Preparation and Manufacture of Polymeric Nanoparticles. Pharm. Res..

[cit29] Karnik R., Gu F., Basto P., Cannizzaro C., Dean L., Kyei-Manu W., Langer R., Farokhzad O. C. (2008). Microfluidic Platform for Controlled Synthesis of Polymeric Nanoparticles. Nano Lett..

[cit30] Mehraji S., DeVoe D. L. (2024). Microfluidic Synthesis of Lipid-Based Nanoparticles for Drug Delivery: Recent Advances and Opportunities. Lab Chip.

[cit31] Bendre A., Bhat M. P., Lee K.-H., Altalhi T., Alruqi M. A., Kurkuri M. (2022). Recent Developments in Microfluidic Technology for Synthesis and Toxicity-Efficiency Studies of Biomedical Nanomaterials. Mater. Today Adv..

[cit32] Dalwadi G., Benson H. A. E., Chen Y. (2005). Comparison of Diafiltration and Tangential Flow Filtration for Purification of Nanoparticle Suspensions. Pharm. Res..

[cit33] Mohr K. (2014). Aggregation Behavior of Polystyrene-Nanoparticles in Human Blood Serum and Its Impact on the in Vivo Distribution in Mice. J. Nanomed. Nanotechnol..

[cit34] Finley J. H. (1961). Spectrophotometric Determination of Polyvinyl Alcohol in Paper Coatings. Anal. Chem..

[cit35] Weischenfeldt J., Porse B. (2008). Bone Marrow-Derived Macrophages (BMM): Isolation and Applications. Cold Spring Harbor Protoc..

[cit36] Oberländer J., Champanhac C., da Costa Marques R., Landfester K., Mailänder V. (2022). Temperature, Concentration, and Surface Modification Influence the Cellular Uptake and the Protein Corona of Polystyrene Nanoparticles. Acta Biomater..

[cit37] Monopoli M. P., Walczyk D., Campbell A., Elia G., Lynch I., Baldelli Bombelli F., Dawson K. A. (2011). Physical–Chemical Aspects of Protein Corona: Relevance to in Vitro and in Vivo Biological Impacts of Nanoparticles. J. Am. Chem. Soc..

[cit38] Monopoli M. P., Åberg C., Salvati A., Dawson K. A. (2012). Biomolecular Coronas Provide the Biological Identity of Nanosized Materials. Nat. Nanotech..

[cit39] Pearson R. M., Juettner V. V., Hong S. (2014). Biomolecular Corona on Nanoparticles: A Survey of Recent Literature and Its Implications in Targeted Drug Delivery. Front. Chem..

[cit40] Shaw J., Pearson R. M. (2022). Nanoparticle Personalized Biomolecular Corona: Implications of Pre-Existing Conditions for Immunomodulation and Cancer. Biomater. Sci..

[cit41] Barbero F., Russo L., Vitali M., Piella J., Salvo I., Borrajo M. L., Busquets-Fité M., Grandori R., Bastús N. G., Casals E., Puntes V. (2017). Formation of the Protein Corona: The Interface between Nanoparticles and the Immune System. Semin. Immunol..

[cit42] Hoshyar N., Gray S., Han H., Bao G. (2016). The Effect of Nanoparticle Size on in Vivo Pharmacokinetics and Cellular Interaction. Nanomedicine.

[cit43] Robertson J. D., Rizzello L., Avila-Olias M., Gaitzsch J., Contini C., Magoń M. S., Renshaw S. A., Battaglia G. (2016). Purification of Nanoparticles by Size and Shape. Sci. Rep..

[cit44] Tan J. S., Butterfield D. E., Voycheck C. L., Caldwell K. D., Li J. T. (1993). Surface Modification of Nanoparticles by PEO/PPO Block Copolymers to Minimize Interactions with Blood Components and Prolong Blood Circulation in Rats. Biomaterials.

[cit45] Miyazawa T., Itaya M., Burdeos G. C., Nakagawa K., Miyazawa T. (2021). A Critical Review of the Use of Surfactant-Coated Nanoparticles in Nanomedicine and Food Nanotechnology. Int. J. Nanomed..

[cit46] Casey L. M., Hughes K. R., Saunders M. N., Miller S. D., Pearson R. M., Shea L. D. (2022). Mechanistic Contributions of Kupffer Cells and Liver Sinusoidal Endothelial Cells in Nanoparticle-Induced Antigen-Specific Immune Tolerance. Biomaterials.

[cit47] Araujo L., Löbenberg R., Kreuter J. (1999). Influence of the Surfactant Concentration on the Body Distribution of Nanoparticles. J. Drug Targeting.

[cit48] Illum L., Davis S. S. (1983). Effect of the Nonionic Surfactant Poloxamer 338 on the Fate and Deposition of Polystyrene Microspheres Following Intravenous Administration. J. Pharm. Sci..

[cit49] Konduru N. V., Jimenez R. J., Swami A., Friend S., Castranova V., Demokritou P., Brain J. D., Molina R. M. (2015). Silica Coating Influences the Corona and Biokinetics of Cerium Oxide Nanoparticles. Part.
Fibre Toxicol..

[cit50] Chen F., Wang G., Griffin J. I., Brenneman B., Banda N. K., Holers V. M., Backos D. S., Wu L., Moghimi S. M., Simberg D. (2017). Complement Proteins Bind to Nanoparticle Protein Corona and Undergo Dynamic Exchange in Vivo. Nat. Nanotechnol..

[cit51] Suk J. S., Xu Q., Kim N., Hanes J., Ensign L. M. (2016). PEGylation as a Strategy for Improving Nanoparticle-Based Drug and Gene Delivery. Adv. Drug Delivery Rev..

[cit52] Gimondi S., Ferreira H., Reis R. L., Neves N. M. (2023). Microfluidic Devices: A Tool for Nanoparticle Synthesis and Performance Evaluation. ACS Nano.

